# Exercise training upregulates intracellular nicotinamide phosphoribosyltransferase expression in humans: a systematic review with meta-analysis

**DOI:** 10.3389/fpubh.2023.1287421

**Published:** 2023-10-26

**Authors:** Xu Sun, Lide Su, Te Bu, Yang Zhang

**Affiliations:** ^1^College of Physical Education, Hunan Normal University, Changsha, China; ^2^School of Humanities, Inner Mongolia University of Technology, Hohhot, China; ^3^Institute of Sports and Health Industry, HEHA CAT Fitness, Changsha, China; ^4^Independent Person, Windermere, FL, United States

**Keywords:** cancer, oxidative stress, mitochondrial, nicotinamide adenine dinucleotide, nicotinamide mononucleotide, nicotinamide phosphoribosyltransferase, physical activity

## Abstract

**Objective:**

Aging is associated with decreased nicotinamide adenine dinucleotide (NAD) levels, which in turn cause dysfunctional mitochondria and indirectly affect a myriad of diseases. Intracellular nicotinamide phosphoribosyltransferase (iNAMPT) serves as a central rate-limiting enzyme in NAD synthesis, making it an indispensable health mediator. This meta-analysis examined the effect of exercise training on the expression of iNAMPT in humans.

**Methods:**

We searched PubMed, Scopus, ClinicalTrials.gov, and the International Clinical Trials Registry Platform for studies published between the inception of the database and July 5, 2023. Using the common-effect model, evidence for the change in iNAMPT following exercise training was synthesized as Cohen’s *d*.

**Results:**

The search yielded five eligible studies. The overall effect size is 0.81, with a 95% confidence interval of 0.55 to 1.07. Therefore, a random adult will have a 71.7% probability that iNAMPT will be up-regulated following exercise training. In general, exercise training resulted in a 1.46-fold increase in iNAMPT. Our probability statistics indicate that subgroups of interest may differ practically. Specifically, there is a 79.3% probability of increased iNAMPT in men, compared to a 69.0% probability in the overall population; young adults have a 75.6% probability of having an increased iNAMPT, whereas aged adults have a 68.7% probability; and, iNAMPT has a 75.1% probability increase after aerobic exercise and a 66.4% probability increase after resistance exercise.

**Conclusion:**

Exercise training is effective for increasing iNAMPT levels in skeletal muscles. This essential enzyme regulates not only cellular energetics but also healthspan. Therefore, exercise should be promoted as a natural slow-aging lifestyle.

## 1. Introduction

The pursuit of longevity by humans has a rich past. Chen Tuan, a Chinese Taoist legendary who lived around 900 A.D., is said to have practiced inner alchemy by drinking wine (cellular dehydration) and sleeping for months [akin to the rate of living theory (1)], which is comparable to dry fasting in today’s longevity community. In 1956, American medical academic Denham Harman first used modern science to explain human aging with his free radical theory (2), which is now supported by empirical evidence (3). Mounting evidence to date suggests that the accumulation of reactive oxygen species damages mitochondria and fundamentally contributes to diseases and aging (4). In the defense against reactive oxygen species, nicotinamide adenine dinucleotide (NAD)-dependent sirtuins emerge as the pivotal regulator of mitochondrial metabolism and homeostasis (5), and nicotinamide phosphoribosyltransferase (NAMPT) stands out as the key catalyst in this process and, more broadly, healthspan and lifespan.

In 1994, NAMPT was first reported as a pre-B-cell colony enhancing factor (6), and as time goes on, accumulating evidence underscores that it is a pleiotropic protein that functions as a growth factor, cytokine, enzyme, and visfatin (7). NAMPT manifests in two forms in humans. The extracellular NAMPT performs a cytokine-like role in the pathological progression of numerous diseases and cancers (7). While the full range of physiological roles of this pleiotropic protein in health and the aging process is a priority area of research, this review focuses on its intracellular form (iNAMPT), a homodimeric class type II phosphoribosyltransferase, which functions as a rate-limiting enzyme of NAD.

In human cells, the mechanism underlying NAD biosynthesis of iNAMPT is well characterized. NAD is an essential energy-sensing metabolite and co-factor for several enzymes that mediate a myriad of signaling pathways, such as metabolism, senescence, cell apoptosis, DNA repair, and gene expression (8). NAD is synthesized through the *de novo*, Preiss-Handler, and salvage pathways. The *de novo* pathway converts L-tryptophan to nicotinic acid mononucleotide via kynurenine and subsequently produces NAD. The Preiss-Handler pathway converts nicotinic acid and nicotinic acid riboside to nicotinic acid mononucleotide via the nicotinic acid phosphoribosyltransferase, then the nicotinamide/nicotinic acid mononucleotide adenylyltransferase converts nicotinic acid mononucleotide to NAD. The salvage pathway is initiated by the addition of a phosphoribosyl moiety to nicotinamide (NAM) from 5-phosphoribosyl-1-pyrophosphate. The key reaction is regulated by iNAMPT, which catalyzes the reversible condensation of NAM to produce nicotinamide mononucleotide (NMN), which is then shared with the nicotinamide/nicotinic acid mononucleotide adenylyltransferase to complete NAD biosynthesis. Of note, nicotinamide riboside also uses the salvage pathway to be converted to NAM or NMN (9), which produces NAD eventually. NAD-dependent enzymes, including sirtuins, ADP-ribosyl cyclases, and poly-ADP-ribose polymerases, cleave NAM from NAD during their reactions, and NAM is subsequently reversed to the salvage pathway. Previous research shows that iNAMPT-KO mice exhibited a dramatic 85% decline in NAD levels, demonstrating the predominance of the salvage pathway in NAD biosynthesis (10). In short, regardless of which precursor (L-tryptophan or vitamin B3) or pathway produces NAD, once NAD is consumed, NAM can be recycled and serves as an intermediate precursor to produce NMN, with iNAMPT playing the master role in the NAD biosynthesis.

Many lifestyle diseases, such as neurodegenerative diseases, metabolic diseases, epigenomic changes, and malignant tumors, can be traced back to mitochondrial dysfunction, which is anchored in NAD depletion thereby mountainous oxidative stress for the body’s energy factory (4). ADP-ribosyl cyclases, for instance, competitively utilize NAD storage during acute immune response and chronically low-grade inflammation, which drains the NAD level for proper mitochondrial function (11). NAD-deficient caused oxygen radical toxicity ultimately results in DNA and mitochondria DNA mutations (12). This chain of events additionally triggers another class of NAD consumers, poly-ADP-ribose polymerases, to use NAD for DNA repair, leaving even less NAD to activate mitochondrial protection via sirtuins-mediated signaling pathways (13). This highlights the importance of sustaining adequate levels of NAD for health and longevity, which inconveniently declines with age (14, 15).

The decline in iNAMPT is a known cause for the free fall of NAD biosynthesis with age. A cross-sectional study shows that young secondary adults (25 years) had about a 1.48-fold higher iNAMPT content compared with aged sedentary adults (70 years) (16). NAD biosynthesis also oscillates in response to nutritional status, which is ultimately affected by age. A recent report from China’s CDC found that the prevalence of inadequate niacin intake increased from 13.0 to 28.40% in females and from 17.75 to 29.46% in males between 1991 and 2018 (17), highlighting a population-wide deficiency of this natural NAD precursor. In the meantime, the aged population tends to be largely deficient in niacin intake (18), and this deficiency is associated with cognitive fragility (19), presumably as a result of low NAD production and, consequently, lower mitochondrial ATP production. As a further complication, niacin absorption decreases with age due to altered energy and protein metabolism or pro-inflammatory changes in the gut. Hence, it is natural to believe that the administration of natural NAD precursors may be a promising strategy for ameliorating the NAD decline associated with aging and diet. Nevertheless, available data indicate otherwise. Human studies reveal that supplementation with L-tryptophan or nicotinic acid did not enhance NAD metabolism (20) and that long-term supplementation may even increase the risk of developing type 2 diabetes (21). Notwithstanding a single observational study, a 12-week supplementation of nicotinamide riboside decreased iNAMPT by 14% in obese and insulin-resistant men (22), an adverse development for NAD and energy metabolism.

There are two viable strategies for combating the age-related NAD decline. Given that NAD replenishment is limited by iNAMPT, boosting iNAMPT expression is the first line of defense. Pharmacological activation of iNAMPT (23) may be contemplated in exceptional cases. In general, this could be accomplished by engaging in regular physical activity regardless of age in healthy individuals. In young adults (< 25 years), endurance-trained athletes had approximately twice as much iNAMPT content as non-obese individuals (24). Data obtained in aged adults (> 65 years) revealed that lifelong football training increased iNAMPT levels by 2.2-fold relative to active untrained compartments (25). The second choice is supplementing cellular sources of NAD precursors, predominantly biosynthetic NMN, making it the focus of research and commercialization. New data underscore the efficacy of biosynthetic NMN in bolstering NAD levels in humans (26), which, if rodent studies are any indication (27), may have positive health effects and promote lifespan.

The emergence of biosynthetic NMN is becoming an increasingly popular topic among longevity enthusiasts. It appears that the general public is more interested in consuming supplements than in engaging in physical activity to achieve perceived health benefits more quickly. Nevertheless, the safety of long-term supplementation with highly potent biosynthetic NMN or NAD precursors in general remains an open question (28). Meanwhile, the molecular underpinnings of the beneficial effects of exercise are far from fully understood, and there are no aggregated data regarding the effect of regular physical activity on iNAMPT expression. Common sense indicates that exercise is the safest option for the general public, and in this regard, an overview of the current understanding is of vital importance. Therefore, this review provides a meta-analysis of the effect of exercise training on iNAMPT expression in humans.

## 2. Methods

### 2.1. Data sources

This study followed the PRISMA 2020 reporting guidelines (29). An electronic search was conducted using PubMed, Scopus, ClinicalTrials.gov, and the International Clinical Trials Registry Platform of the World Health Organization from the earliest date available to 5th July 2023. We used the following search term pairing in the title and abstract: (aerobic exercise OR endurance exercise OR resistance exercise OR training) AND (nicotinamide phosphoribosyl transferase OR nicotinamide phosphoribosyltransferase OR NAMPT). After filtering the records, we conducted a snowball search to identify additional studies by backward searching the reference lists of eligible studies and forward searching (via Google Scholar) studies citing them.

### 2.2. Eligibility criteria

Search results were imported to Endnote 21 for reference management. The records were initially screened automatically using the following exclusion criteria: animal studies; human studies involving children; non-English publications; or, review studies. The remaining records were then evaluated based on the following inclusion criteria: reported outcome of interest; reported pre- and post-training outcome; and, data can be calculated for effect size. Two researchers conducted these procedures independently, and disagreements or uncertainties regarding eligibility were resolved through discussion. Of note, the corresponding authors of two studies were contacted for additional information regarding their raw mean difference data. These studies were removed from the final list after two attempts to contact them via email were unresponsive.

### 2.3. Outcome

The outcome measure in this study is iNAMPT in skeletal muscles. Data reporting that permits effect size calculation was eligible for inclusion. Outcome data were extracted from figures using the following method: the figure of interest was imported into Adobe Photoshop, and then the ruler tool was used to measure the figure elements (i.e., length and scale), with the resultant measurement calculated in an Excel spreadsheet. Two researchers independently extracted data, and their measurements were 99% concordant. The measured data, such as those from Costford et al. (24) exhibit an extremely high degree of accuracy (100%) when compared to the reported data (i.e., an increase of 127%). Of note, the iNAMPT of the study by Lanza et al. (30) was extracted from the supplementary materials of the study by Johnson et al. (16). The data that support the conclusions of this study are available on figshare.^1^

### 2.4. Data synthesis and analysis

Studies that examined different stages of life were considered independent research, and effect sizes were determined for each age cohort. This meta-analysis adopts Rosenthal’s recommendation (31) to use a conservative estimate of the pre-post correlation coefficient, 0.7, in the calculation of effect size from mean change scores of paired-sample studies. The robustness of the results from any imputed correlation coefficients was evaluated via sensitivity analysis. In experimental studies, Hedges’ *g* is a preferred method for reporting the effect size of small sample sizes. In a meta-analysis, however, Hedges’ *g* may lead to more biased meta-estimates than Cohen’s *d* (32). The effect size reported in this study is therefore Cohen’s *d*.

This meta-analysis was conducted using the meta package version 6.5–0 in the RStudio version 2023.06.0 Build 421. The estimation of between-study variance was fixed using the restricted maximum-likelihood method. Due to the limited number of eligible studies, the effect sizes of individual studies were synthesized into a common effect size (33). The *I*^2^ statistic (34) was computed to assess the contribution of heterogeneity to the aggregated effect size. *I*^2^ values of 25, 50, and 75% represent low, moderate, and high inter-study heterogeneity relative to the intra-study variance, respectively.

In addition to the aggregate effect size, this study examined potential confounding moderators. Subgroup analyses include age (i.e., < 30 years old as young vs. > 50 years old as aged), sex (i.e., male-only vs. male and female), research design (i.e., independent sample vs. paired sample), and mode of exercise training (i.e., aerobic exercise vs. resistance exercise). Subgroup heterogeneity was determined using Cochran’s *Q* statistic. Of note, the experiment conducted by Brandauer and colleagues (35) could be also coded as a paired-sample study. In this meta-analysis, it was coded as an independent-sample study (see also discussion of its practical significance). Its paired-sample and independent-sample effect sizes are 1.183 and 1.516, respectively.

We also calculated the weighted average fold change of iNAMPT following exercise training. Because one included study (16) cannot be used to calculate its original fold change, we removed it and recalculated the weight of each study using the procedures described for this meta-analysis. We then calculated the weighted average fold change by combining the individual study fold changes.

Given that the eligible studies encompass both single-arm and randomized controlled trials, we used both RoB 2 (36) and ROBINS-I (37) to evaluate the certainty of evidence. Two researchers independently applied the tools to each of the included studies, and any disagreements in risk of bias assessments were resolved to reach a consensus. The domains were then combined using the generic risk-of-bias framework of the R-based robvis tool (version 0.3.0) (38). Both Egger’s regression test and a visual inspection of the funnel plot (39) were used to assess publication bias.

To facilitate broad dissemination beyond the research community, this meta-analysis effect size is interpreted based on the common-language effect size. The equation (40) used for the conversion from Cohen’s *d* is as follows:


$$\mathrm{Common language effect size}=\Phi \left(\frac{d}{\sqrt{2}}\right)\text{,}$$

where, Ф is the normal cumulative distribution function.

## 3. Results

### 3.1. Included studies

**Figure 1:**
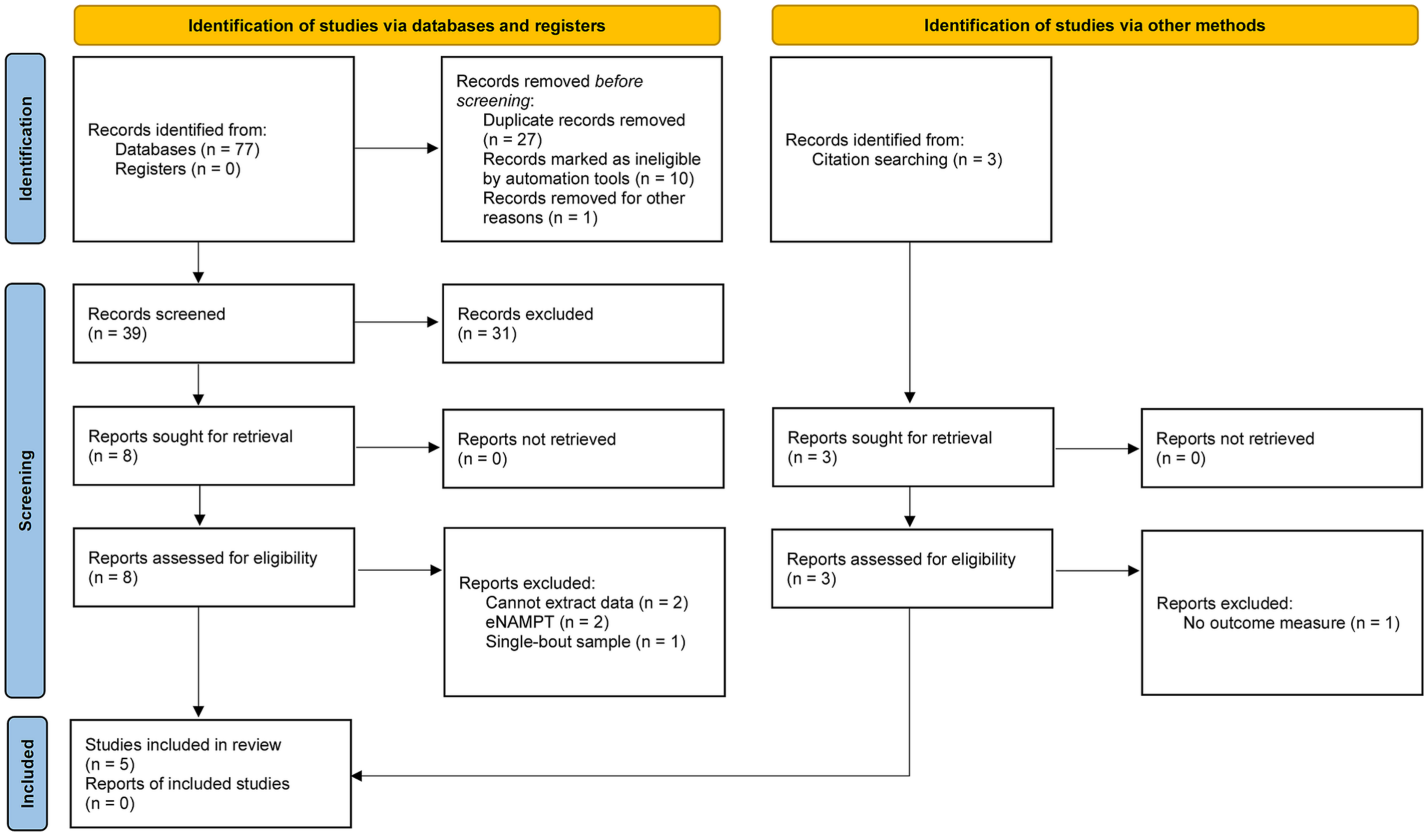
PRISMA flow diagram.

As of 5 July 2023, 77 records were located from the search strategy, which is depicted in Figure 1. Following screening, five studies met all inclusion criteria (16, 24, 30, 35, 41). Table 1 summarizes the general characteristics of the included studies. All studies enrolled healthy participants without major chronic health conditions that could confound the outcome measure. Both male and female participants contributed to the evidence and corresponding conclusions. Two studies are randomized controlled trials, two studies are single-arm trials, and as aforementioned, one study could be considered a pair-sample or independent-sample study. Given that this field of research necessitates invasive muscle biopsies and supervised exercise training, it is understandable that all studies recruited small sample sizes. Aerobic exercise is the dominant training mode, and the training duration was as short as 3 weeks.

**Table 1:** Characteristics of included studies.

Source (citation)	Baseline, exercise-group participants				Study design	Exercise intervention	
	*N*, sex	Age (yr)	BMI (kg/m^2^)	V̇O_2max_ (ml/kg/min)		Mode	Loading
Brandauer (35)	8 M	25.0	24.6	49.0	I	A	3 wks: 1–2 h/session at 70–85% peak workload, 15 sessions
Costford (24)	10 M	< 30^1^	–	–	P	A	3 wks: 30–60 min at 75–85% V̇O_2max_ or 50 min at 70% V̇O_2max_ per session × 13
Johnson (16)(young)	6 M/5F	24.9	24.0	29.5	I	A	8 wks: 60 min at 65% V̇O_2max_ per session, 3–5 session/wk
Johnson (16) (aged)	5 M/5F	70.0	27.9	17.8	I	A	8 wks: 60 min at 65% V̇O_2max_ per session, 3–5 session/wk
Lamb (41)	6 M/10F	59.0	31.7	–	P	R	10 wks: 10–12 repetitions × 3 sets of whole-body exercise, 2 session/wk
Lanza (30) (young)	6 M/5F	26.0	22.7	51.6	I	A	4 yrs.: >1 h, 6 times/wk., self-reported
Lanza (30) (aged)	6 M/4F	65.4	24.4	39.4	I	A	4 yrs.: >1 h, 6 times/wk., self-reported

Data are expressed as means. Subgroups were evaluated as independent datasets. A, aerobic exercise; F, female; M, male; I, independent sample; R, resistance exercise; P, paired sample. ^1^Based on the inclusion criteria, participants’ ages ranged from 18 to 30.

**Figure 2:**
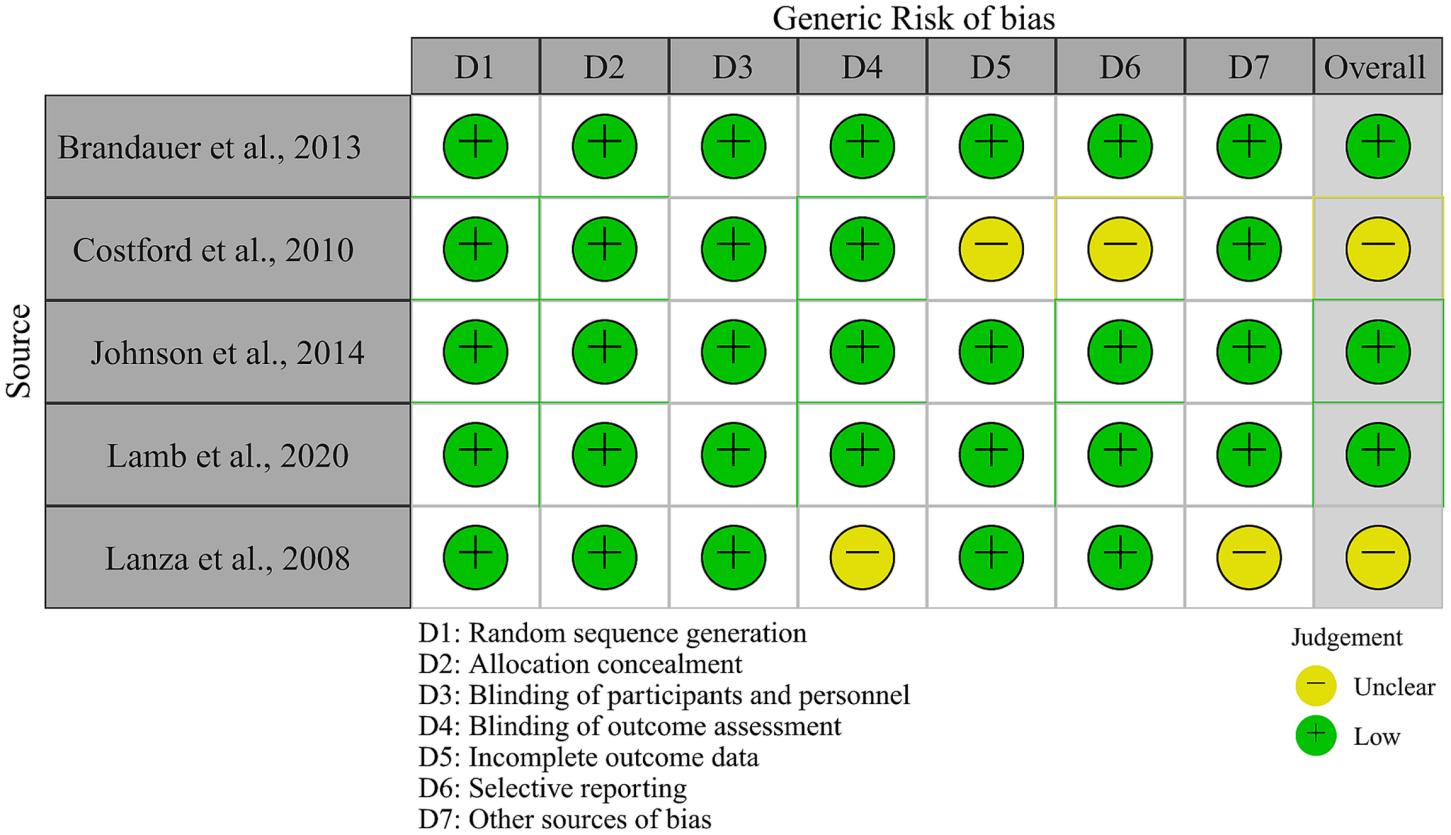
Risk-of-bias assessment of included studies.

Figure 2 depicts the certainty of evidence. These research, in general, provided quality evidence from well-designed experiments. There are however two concerns. For Costford and colleagues’ study (24), there exists an attrition bias. Specifically, 13 participants served as their own controls. In the post-training evaluation, iNAMPT expression decreased by three individuals, but not other measures (e.g., iNAMPT RNA). It is unknown whether the sample difference was due to an issue from analytic procedures, selective reporting, or other factors. Therefore, this issue represents a moderate risk of bias.

A self-reporting bias exists in Lanza and colleague’s study (30). In their investigation, the levels of physical activity (i.e., exercise training) over the past 4 years were determined using a questionnaire. The problem there is twofold. Whether or not participants were concealed regarding the purpose of the study before the survey, recall of physical activity history presents inaccuracy regarding exercise duration and frequency (42). If participants were aware of the purpose of the study and over-reported their exercise history, this would result in a misclassification bias, invalidating their conclusions and impacting the effect size in this meta-analysis. We include their study in this meta-analysis because it presents a rare set of long-term training effects (two other long-term soccer training studies are excluded from this meta-analysis because the effect size could not be calculated). Self-reporting bias and possibly misclassification bias pose a moderate or critical risk of bias, which readers should be aware of.

### 3.2. Exercise training enhances intracellular nicotinamide phosphoribosyltransferase expression

**Figure 3:**
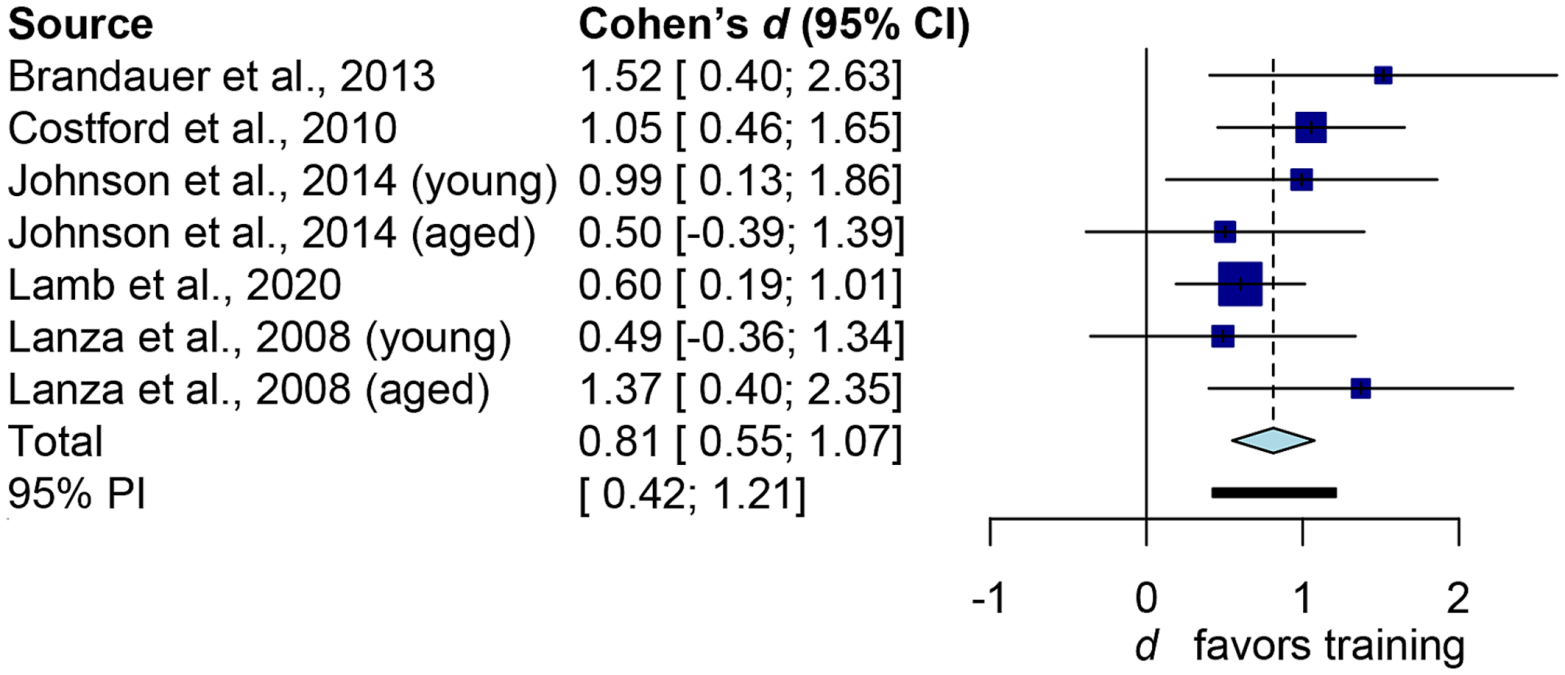
Forest plot of human’s iNAMPT expression following exercise training. The squares represent the results of independent datasets, with the size of each square proportional to the dataset’s weight. The horizontal line represents the 95% confidence interval. The diamond represents the pooled result. The bold horizontal line represents the 95% predictions.

Figure 3 depicts the main outcome. The aggregated *d* is 0.81 (95% confidence interval: 0.55 to 1.07), which conventionally represents a large effect size. The common-language effect size was calculated to facilitate non-clinical practitioners and the general public in interpreting the results. Cohen’s *d* of 0.81 yields *A* value of 0.717, indicating a 71.7% probability that a randomly selected man or woman will have higher iNAMPT expression following exercise training than a randomly selected physically inactive adult. Overall, exercise training led to a 1.46-fold increase in iNAMPT. We then examined how imputation impacts our results. By assuming *r* = 0 in paired-sample studies, the pooled result shifted slightly to the right (larger): *d* = 0.89, 95% confidence interval = 0.54 to 1.24. This difference, however, is trivial from both a theoretical perspective (*d* ≥ 0.8 as a large effect size) and a probabilistic standpoint (71.7% vs. 73.5%). Thus, sensitivity analysis confirms that imputations of correlation coefficients have little influence on the practical inference of the results. Notwithstanding the *I*^2^ statistic yielded a 0% value, indicating that there was no inter-study heterogeneity. Table 2 presents the results from subgroups of interest, which are practically interpreted in the discussion.

**Table 2:** Subgroup analyses exploring moderators that influence iNAMPT expression following exercise training.

Moderator	No. of dataset	Sample size	Cohen’s *d* (95% CI)	CLES (95% CI; %)	Between-group *p*
Sex					
Male only	2	18	1.16 (0.63 to 1.68)	79.3 (67.2 to 88.3)	0.138
Male and female	5	58	0.70 (0.40 to 1.00)	69.0 (61.1 to 76.0)	
Age					
Young	4	50	0.98 (0.58 to 1.37)	75.6 (65.9 to 83.4)	0.282
Aged	3	26	0.69 (0.34 to 1.03)	68.7 (59.5 to 76.7)	
Research design					
Paired sample	2	26	0.75 (0.41 to 1.09)	70.2 (61.4 to 78.0)	0.555
Independent sample	5	50	0.91 (0.49 to 1.32)	74.0 (63.6 to 82.5)	
Training mode					
Aerobic exercise	6	60	0.96 (0.61 to 1.30)	75.1 (66.7 to 82.1)	0.195
Resistance exercise	1	16	0.60 (0.19 to 1.01)	66.4 (55.3 to 76.2)	

CLES, common-language effect size.

### 3.3. Publication bias

**Figure 4:**
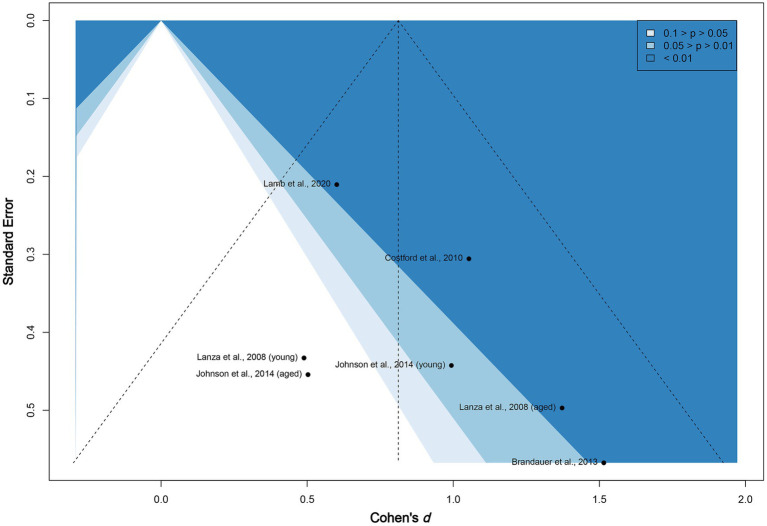
Funnel plot of included studies. The vertical line marks the overall Cohen’s *d*. The figure is contour enhanced using conventional benchmarks for statistical significance.

The funnel plot given in Figure 4 does not show subjective asymmetrical scatter along the vertical line. We then examined the contour-enhanced plot. The included studies are located in both non-significant (white, *p* > 0.05) and highly statistically significant (dark blue, *p* < 0.01) regions, reinforcing that publication bias among the existing published experiments appears to be low. In addition, the *value of p* of Egger’s test for asymmetry is non-significant (two-tailed *p* = 0.22). We conclude that neither material selection nor publication bias exists in this meta-analysis.

## 4. Discussion

Earth provides humanity with a habitable environment, and one of its fundamental elements is oxygen. Mitochondria use oxygen to sustain oxidative phosphorylation, the TCA cycle, and glycolysis to produce ATP; however, this process imposes an oxidative “tax.” Oxidation is a natural trigger for epigenomic changes, and aging hastens this process. It is known that exercise confers resistance to mitochondrial defects induced by oxidative insults, in part through boosting NAD biosynthesis. Here, we provide an overview of existing human studies that investigated iNAMPT expression following exercise training. In line with animal studies, exercise can effectively up-regulate iNAMPT expression in human skeletal muscles. When adults engage in structured physical activity, there is a 71.7% probability of superiority or a 1.46-fold increase in iNAMPT. Although our outcome measure only confirms one critical change in the intermediate process of NAD biosynthesis, existing human studies indeed show that exercise-induced iNAMPT up-regulation can increase NAD (41), NAD-dependent sirtuin deacylases (30), antioxidants (16), and senescent signaling pathways (25, 43). Therefore, this meta-analysis validates the cellular crosstalk between exercise training and metabolism, epigenetics, and aging via iNAMPT.

Although this study addresses the extent to which exercise training exerts cellular effects in skeletal muscles, the precise mechanism by which iNAMPT is up-regulated and maintained remains unclear. From a systematic perspective, the simplest explanation for the mechanism would be aerobic and resistance exercises both promote skeletal muscle hypertrophy (44). Exercise training increases the genetic expression of mitochondria and electron transport chain activities (45), hence an increase in mitochondrial contents in skeletal muscles would be expected to be accompanied by the up-regulation of iNAMPT (24). On the other hand, this positive adaptation could theoretically be reversed if exercise training is discontinued for an extended period, and this may be the most plausible explanation for the down-regulation of iNAMPT and mitochondrial redox potential due to age-associated progressive muscle atrophy. In the meantime, the localized expression and function of iNAMPT in diverse tissues (46), such as smooth muscle, myocardium, and hypothalamus, remind us that the molecular mechanism underlying the adaptation to exercise training is far from clear. Therefore, tissue- and/or cell-specific kinetics of NAMPT expression in humans should be clarified in future studies.

This systematic review further provides the first quantitative evidence on a few topics of interest that disparate conclusions are partially attributable to different statistical inferences. Based on Cochran’s *Q* statistic in the conventional meta-analysis, we shall conclude that there is no sub-group difference and that the effect of exercise training is therefore univariate. However, when interpreting the results using probability distribution theory, we are compelled to suggest that the context appears to be more complex and that no rash conclusions should be drawn. In particular, three subgroups of interest merit clarification in the future. First, there is a nearly 10% probability difference between studies that only included men and those that included both sexes, indicating that men may be more responsive to exercise training. Using common sense, it appears that this is true. As aforementioned, the up-regulation of iNAMPT may be directly linked to an increase in mitochondrial contents during muscle hypertrophy. In this regard, men typically have bigger total muscle mass and a larger cross-sectional area than women (47), with an increase in mitochondrial content concurrent with a rise in cross-sectional area (48). This difference in baseline may lead to additional muscular adaptations in men following exercise training. Apart from the physiological difference, the discrepancies may also be attributed to modern lifestyle factors. The enhanced level of female work participation is undoubtedly a milestone in the realm of gender equality. Yet, female labor involvement often falls within the category of office work, hence leading to a greater prevalence of sedentary behaviors and longer working hours among modern women. From a sociocultural standpoint, there also tends to be a lower level of female participation in physical activities (49). Both conditions are not conductive to maintaining iNAMPT levels. Notwithstanding the need for further clarification of these distinctions, cellular response evidence suggests that it is advisable for women to allocate a greater amount of leisure-time physical activity in order to optimize iNAMPT expression.

Second, the nearly 7% difference in probability between young and aged adults may indicate biological changes in aging. Again, this may indicate a relative tissue-specific change, such as muscle atrophy. This may also be attributable to the absolute loss of mitochondrial contents and oxidative capacity associated with aging (50). Nevertheless, the present data support the notion that exercise training offers an optimal method for enhancing the pathways of NAD production for healthspan. In fact, NAD production in aged adults increased to comparable levels in young adults following exercise training (41), suggesting the progressive decline in NAD biosynthesis during aging is reversible and iNAMPT is the master link in this regard.

Third, the nearly 9% difference in probability between aerobic exercise and resistance exercise may not be a random occurrence. There may be a dose-dependent association between iNAMPT expression and the mode/intensity of exercise training, and mode and intensity tend to be interrelated. Prolonged moderate-intensity exercise transfers more energy demands to the glycolytic pathway, which requires adequate NAD production to activate the TCA cycle and sirtuins pathways in mitochondria. This further necessitates rapid recycling between NAM and NAD, resulting in a metabolic adaptation characterized by increased iNAMPT expression. Contrarily, sub-maximal resistance exercise or isotonic contractions operate on ATP supplies derived from the breakdown of phosphocreatine, and the energy nature has a lower demand for NAD production (51). Clear differences therefore exist between aerobic and resistance exercise, thus making the preferred mode of exercise the former. Despite this theoretical benefit, we recommend a combination of aerobic and resistance exercise for full-spectrum health. The primary justification for this recommendation derives from an experimental study showing that exercise training increased iNAMPT expression in trained skeletal muscle, but not in untrained skeletal muscle (11). Our interpretation is that training should involve as many muscle groups as possible to maximize iNAMPT expression in the entire body. Additionally, resistance exercise provides many benefits for the quality of independent living during aging (52). To counteract the effects of aging, the optimal combination may include more aerobic exercise days, such as four to five per week, combined with resistance and balance training to reconstitute NAD levels and delay functional declines associated with aging.

While this meta-analysis strives to elucidate the nexus between exercise training and iNAMPT regulation and their implications for aging, it also serves as a crucial reminder that good health is never a young privilege. Research results indicate that endurance-trained athletes exhibit approximately double the expression of iNAMPT compared to young non-obese individuals, while obese and T2D patients demonstrate drastically decreased iNAMPT expression (24). This suggests that increasing iNAMPT levels can be achieved as a predictable benefit of an active lifestyle. Unfortunately, we are witnessing the most intense polarization between global GDP expansion and the level of physical inactivity in recorded human history. The findings of a recent demographic research revealed 67.2% of the Chinese population to be physically inactive and is associated with a rise in noncommunicable diseases (53), underscoring our view that engaging in exercise training is a contributing factor to maintaining good health. According to data from the WHO global health observatory data repository, the average duration of moderate to vigorous physical activity was approximately 35.5 min per day for men and 32.0 min per day for women in 2012 (49). This is problematic. Not only is there evidence of a global decline in physical activity, but the favorable results revealed in this meta-analysis are contingent upon engaging in longer duration (i.e., 60 min each session) or more structured exercise training. Hence, only by adopting an active lifestyle, characterized by chronic participation in multi-component physical activities such as recommended in the current WHO guideline (54), could people of all ages maintain NAD levels and improve the likelihood of healthspan.

**Figure 5:**
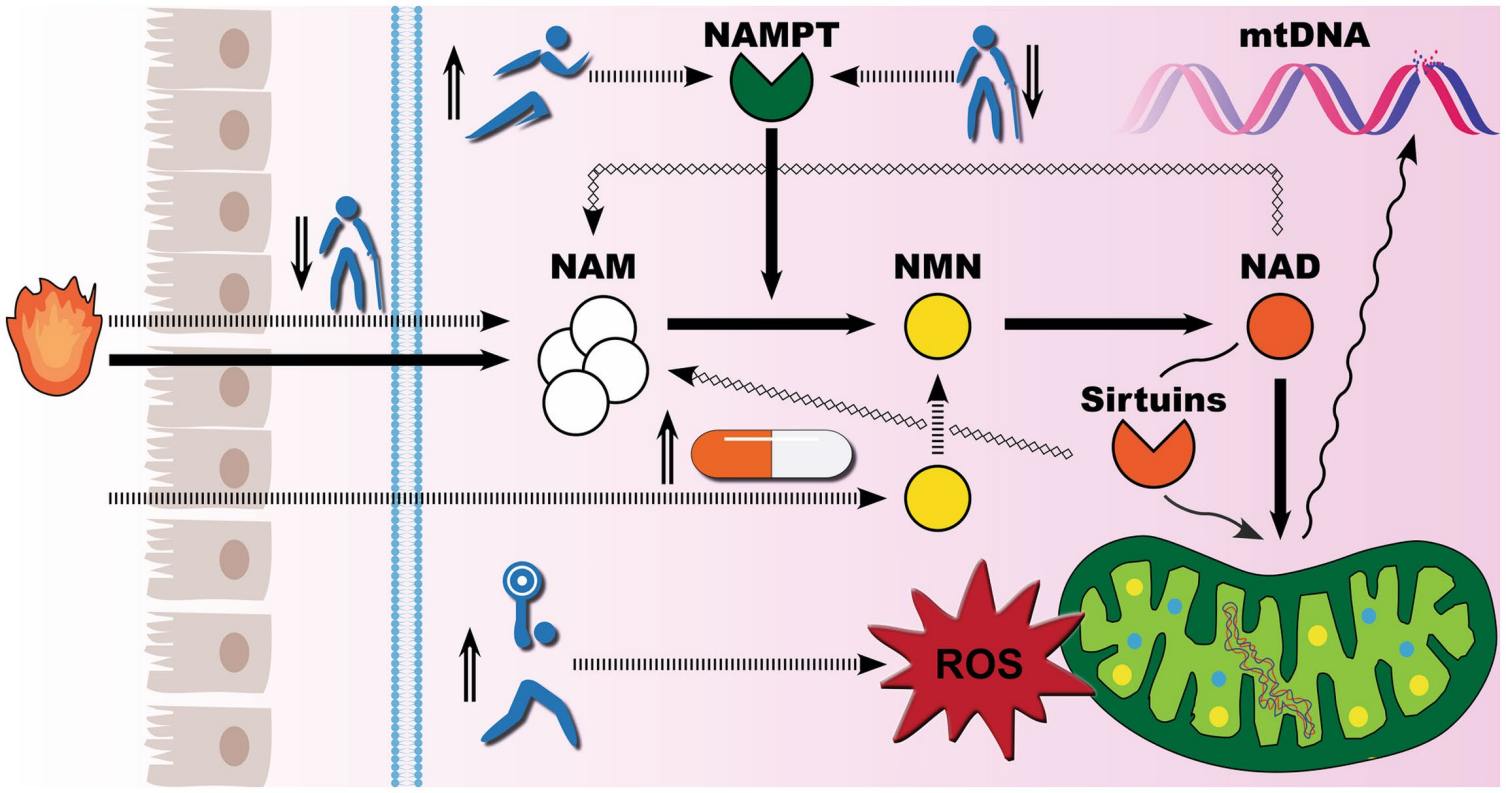
Exercise meets supplements for healthy aging: competing demand for NAD biosynthesis. Mounting evidence suggests that mitochondrial dysfunction is the underlying cause of the rise in noncommunicable diseases. To defend against environmental insults, healthy levels of nicotinamide adenine dinucleotide (NAD) must be maintained. There are three biosynthetic pathways for NAD production in human cells, with the salvage pathway playing the predominant role. Socioeconomic status, aging-associated changes in cellular metabolism, and chronic low-grade inflammation can all result in niacin deficiency and sub-clinical nicotinamide (NAM) levels, thereby compromising the robustness of the salvage pathway for NAD metabolism. Meanwhile, the salvage pathway exclusively depends on the intracellular nicotinamide phosphoribosyltransferase (iNAMPT) to catalyze the conversion of NAM to nicotinamide mononucleotide (NMN). iNAMPT inconveniently declines with age, further impeding NAD production. When NAD recycling cannot meet its demand, such as NAD-dependent sirtuin 3 activation for mitochondrial DNA (mtDNA) repair, aberrant cellular environments may eventually result in mtDNA breakage and mutations. There are two primary strategies to boost NAD biosynthesis. The natural way to induce iNAMPT expression is through exercise training, while the modern way is to provide NAD precursors such as biosynthetic NMN. Of note, exercise training also induces a dose-dependent spike in the production of reactive oxygen species. As global GDP reaches a certain level, people become more health conscious, staging biosynthetic NMN and exercise in the spotlight for life extension.

On the basis of the available evidence, exercise training alone is an effective regenerative method for reversing the progressive decline in iNAMPT expression in skeletal muscles, and aged adults may even attain NAD levels comparable to those of young adults. While there is no comparable investigation of iNAMPT or NAD in skeletal muscles following supplementation with NAD precursors, several studies have shown that supplementation with biosynthetic NMN increases circulating NAD levels. A recent study found that biosynthetic NMN improved circulating NAD levels in a dose-dependent manner, with NAD levels quadrupling after 30 days of supplementation with 900 mg of biosynthetic NMN (55). As illustrated in Figure 5, it appears that NMN supplementation offers the least resistance to boosting NAD levels. The crucial difference between exercise training and biosynthetic NMN is that exercise training, at the right range of intensity, also promotes healthy oxidative stress that enhances mitochondrial innate resistance to environmental insults (56), while the benefits of biosynthetic NMN are entirely NAD-driven, with sufficient NAD levels to activate sirtuins signaling pathways for proper mitochondria DNA repair during stress. Furthermore, it remains debatable whether ultra-high NAD storage is entirely beneficial for lifespan. NAD is used in all cells, including cancer cells to produce energy. One hallmark of the Warburg effect is tumor glycolysis (57), and recent animal data confirm concerns (58) that popular forms of NAD precursor exhibited a proliferative influence on tumor growth (59, 60).

That said, we are not lukewarm about new biosynthetic NAD precursors, let alone among the denier camp. We are eager to examine their short-term effect on cancer treatment, as a recent study suggested (60). In Zhang and colleagues’ animal model, low-dose NMN supplementation promoted cancer cell proliferation whereas high-dose NMN supplementation led to cancer cell apoptosis. The treatment of newly diagnosed cancer patients who regularly engage in physical activity, in our opinion, should place special focus on inhibiting iNAMPT or NAMPT expression in general. The rationale is obvious. Long-term exercise could maintain iNAMPT expression in skeletal muscles shown here and possibly in other tissues. Under healthy conditions, it is a pro-health and pro-longevity enzyme, whereas under cancerous ones, it may promote NAD biosynthesis and fuel cancer cells indiscriminately. In conjunction with high-dose NMN supplementation, this may represent a new line of therapy, particularly for athletes.

Finally, we find as many questions as we answer. Exercise training includes various forms, and this meta-analysis is based on data yielded from aerobic and resistance exercise. There is no data to support or deny if low-intensity exercise, such as the popular walking, yoga, pilates, hiking, isometric exercise, or Tai Chi, may yield a similar magnitude of benefits shown here. This remains an important question to be answered as walking in certain cultures, such as China, is a very popular form of daily physical activity among aged people. Meanwhile, the aerobic exercise prescribed among the included studies is moderate-intensity type, and fitness communities nowadays often favor high-intensity interval type training. Whist higher-intensity exercise may stimulate similar or even better iNANMPT expression in the short run, it also introduces more oxidative stress. As reported by Sureda and colleagues, exercise below 90% maximal oxygen consumption resulted in a mild and non-significant increase in reactive oxygen species production, whereas exercise above 90% maximal oxygen consumption increased reactive oxygen species production by 1.4-fold relative to resting states (61). Practitioners with limited scientific training might solely focus on the short-term advantages. It appears that moderate-intensity exercise could lead to a mirage of benefits but no overwhelming reactive oxygen species response for mitochondrial up-regulation of the stress response. The biological significance of reactive oxygen species overload from exercise and whether or not an excess of these species would cancel out the beneficial effects of exercise on normal mitochondrial function remain an uncharted area, and further investigations are awaited.

In conclusion, this meta-analysis demonstrates that aerobic and resistance exercises are effective in increasing iNAMPT expression in skeletal muscles and that this link plays a pivotal role in NAD metabolism, which is gaining increasing attention as a therapeutic target for the treatment of many noncommunicable diseases and chronic conditions. As the global population structure is rapidly transitioning toward fewer working and more retirement cohorts, preparing for the socioeconomic shifts associated with an aging population is essential to fulfilling the pledge of the 2030 Agenda that “no one will be left behind.” This research is an integral part of global efforts to promote healthy aging and recommends a natural and effective strategy from the sports sector.

## Data availability statement

The datasets presented in this study can be found in online repositories. The names of the repository/repositories and accession number (s) can be found at: https://doi.org/10.6084/m9.figshare.23998203.v1.

## Author contributions

XS: Data curation, Formal analysis, Validation, Writing – original draft. LS: Data curation, Formal analysis, Validation, Writing – original draft. TB: Resources, Writing – review & editing. YZ: Conceptualization, Data curation, Formal analysis, Methodology, Supervision, Visualization, Writing – review & editing.

## Footnotes

^1^https://doi.org/10.6084/m9.figshare.23998203.v1
